# A novel spot mutation leading to sialidosis type 1-myoclonus syndrome and optical coherence tomography findings

**DOI:** 10.5935/0004-2749.2022-0069

**Published:** 2023-03-08

**Authors:** Selma Meşen, Muhammed Batur, Muhammet Derda Ozer

**Affiliations:** 1 Eye Clinic, Türkoğlu Dr. Kemal Beyazıt Public Hospital, Türkoğlu, Turkey; 2 Ophthalmology Department, Faculty of Medicine, Van Yuzuncu Yil University, Tusba, Turkey; 3 Ophthalmology Department, Faculty of Medicine, Bandırma Onyedi Eylül University, Balikesir, Turkey

**Keywords:** Mucolipidosis, Myoclonus, Sialidosis type 1, Tomography, optical coherence, Gene NEU1, Mucolipidoses, Mioclonia, Sialidose tipo 1, Tomografia de coerência óptica, Gene NEU1

## Abstract

This report presents the optical coherence tomography findings and a new *NEU1* mutation in bilateral macular cherry-red spot syndrome associated with sialidosis type 1. A 19-year-old patient with a macular cherry-red spot underwent metabolic and genetic analyses supported by spectral-domain optical coherence tomography. Fundus examination revealed bilateral macular cherry-red spot. Spectral-domain optical coherence tomography revealed increased hyperreflectivity in the retinal inner layers and the photoreceptor layer in the foveal region. The genetic analysis detected a new *NEU1* mutation, which caused type I sialidosis. In cases with a macular cherry-red spot, sialidosis should be included in the differential diagnosis, and *NEU1* mutation should be screened. Spectral-domain optical coherence tomography alone is not sufficient in the differential diagnosis because childhood metabolic diseases may exhibit similar signs.

## Abstract (pt)

Neste artigo, objetivamos apresentar os achados da tomografia de coerência óptica em uma nova mutação detectada no gene NEU1 em um caso de síndrome macular vermelho-cereja bilateral associada à sialidose tipo 1. Um paciente de 19 anos com um achado de mancha macular vermelho-cereja foi submetido a análises metabólicas e genéticas, apoiadas por imagens de tomografia de coerência óptica de domínio espectral (SD-OCT). Ao exame de fundo de olho, foi observada uma mancha macular vermelho-cereja bilateral. Nas imagens de SD-OCT, observou-se hiper-refletividade nas camadas internas da retina e na camada fotorreceptora na região foveal. Foi realizada uma análise genética e uma nova mutação foi detectada no gene NEU1, resultando em sialidose tipo 1. Nos casos em que é detectada uma mancha vermelho-cereja na mácula, o diagnóstico diferencial de sialidose deve ser feito e mutações do gene NEU1 devem ser rastreadas. A SD-OCT por si só não é suficiente para o diagnóstico diferencial, porque achados de aparência semelhante podem se manifestar em casos de doenças metabólicas da infância.

## INTRODUCTION

Sialidosis is a lysosomal storage disease with autosomal recessive inheritance. It is characterized by the accumulation of sialic acid-containing oligosaccharides in tissues where the activity of neuraminidase enzyme is impaired due to a mutation in the neuraminidase gene (*NEU1*). Sialidosis is divided into two subtypes according to clinical features and prognosis. Type 1 sialidosis, a late-onset and milder form, is known as the cherry-red spot (CRS) myoclonus syndrome and progresses with visual impairment, macular CRS, myoclonus, ataxia, and seizures^(1,2)^.

CRS is a clinical sign that results from retinal thickening and diminished transparency in the posterior pole. CRS is associated with certain metabolic storage disorders, central retinal artery occlusion, orbital trauma, and ischemia. It was also reported in cases of quinine, carbon monoxide, methanol, and dapsone toxicity.

Herein, we present the spectral-domain optical coherence tomography (SD-OCT) findings of a patient with type 1 sialidosis with macular CRS of both eyes. To our knowledge, this is the first case presented from our country in terms of ophthalmological data in type 1 sialidosis with a novel *NEU1* mutation.

## CASE REPORT

**Figure 1:**
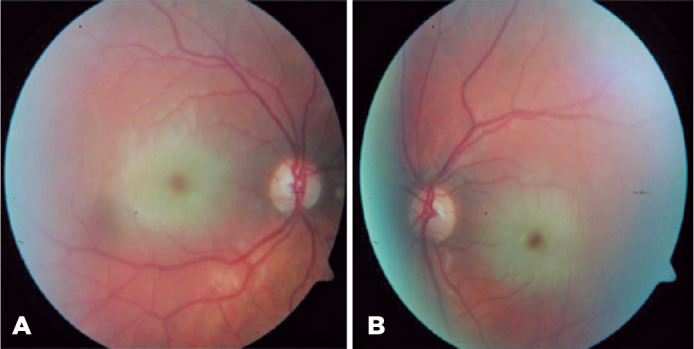
Fundus examination revealed a cherry-red spot in both eyes of thespatient.

**Figure 2:**
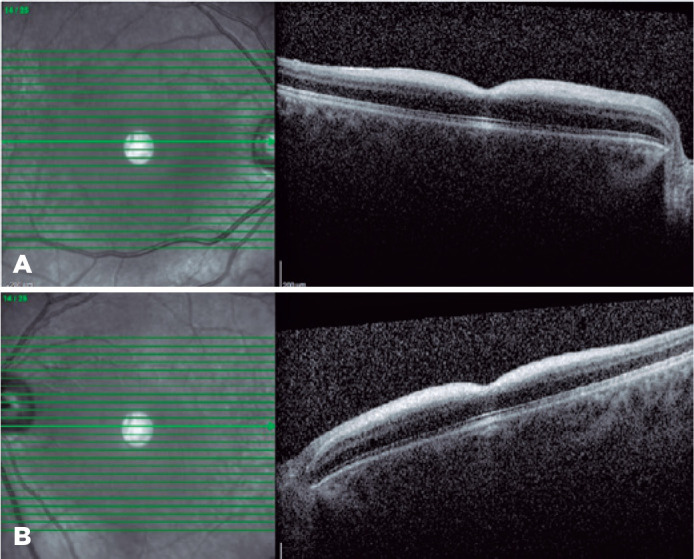
Macular scan of spectral-domain optical coherence tomography, showing increased reflectivity of the inner retinal layer, and apparent hyperreflectivity of the photoreceptor layers in the foveal region of the right and left eyes.

**Figure 3:**
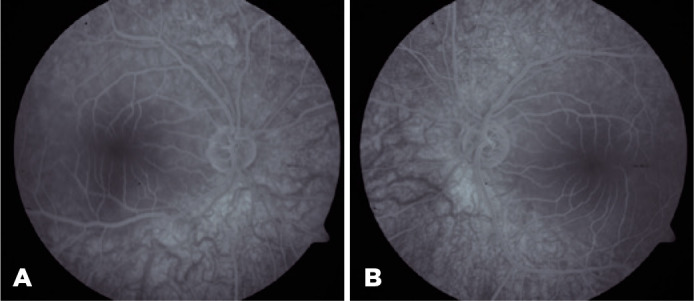
Fundus fluorescein angiography recirculation phase photos. Note the slight shading and mild hypofluorescence with a blurred appea-rance at the vessel borders.

A 19-year-old female patient presented to our clinic with complaints of bilaterally decreased vision for the past 8 years. These complaints had been worsening within 5 years, which were thought to be fatigue, tremor, ataxia, and myoclonic epilepsy after simple daily activities. Her siblings had no history of neurological and ophthalmic diseases. Her best-corrected visual acuity was 20/125 on the right and 20/200 on the left. Slit-lamp examination revealed clear cornea in both eyes and multiple punctate opacities in both lenses. In the fundus examination, bilateral macular CRS was observed with its characteristic red fovea and pale macula (Figure 1). SD-OCT (Heidelberg Engineering, Heidelberg, Germany) imaging revealed hyperreflectivity of the inner retinal layers and increased hyperreflectivity on the photoreceptor layer in the foveola region (Figure 2). In the fundus fluorescein angiography (FFA), slight shading and mild hypofluorescence with a blurred appearance at the vessel borders were observed in the macula (Figure 3). The patient was referred to the neurology department, and various analyses were performed (Table 1).

**Table 1:** Disorders that have role in cherry-red spot etiopathogenesis and tests performe

Disorders	Enzyme deficiency	Abnormal findings	Our patient’s value/(normal range)
Niemann-Pick	Sphingomyelinase	Hepatosplenomegaly Mental disorder Myoclonus Peripheral neuropathy	4,3 µmol/L/h (n>0.9)
GM 2 type I (Tay-Sachs)	Hexosaminidase B-alpha subunit	Hepatosplenomegaly Mental disorder Myoclonus Spasticity	78.30 nmol/mL/h (n=24.00-130.00)
GM 2 type II (Sandhoff)	Hexosaminidase A and B-beta subunit	Hepatosplenomegaly Mental disorder Myoclonus Spasticity	379.23 nmol/mL/h (n=30.00-765.00)
Krabbe	B-Galactocerebroside	Peripheral neuropathymental disorder SpasticityUrinary sialic acid excretion	4.25 µmol/h/L (n>0.5)
GM1 gangliosidosis	B-galactosidase	Rough face view Hepatosplenomegaly Kardiopathy Mental disorder	46.74 nmol/mL/h (n=16.10-115.00)
Galaktosialidozis	B-galactosidase and sialidase	Hepatosplenomegaly Mental disorder Myoclonus Kardiopathy	B-galactosidase was normal
Metachromatic leukodystrophy (lipidoses)	Arylsulfatase A	Peripheral neuropathy Mental disorder	81 nmol/s/mg pr (n=50-990)

The genomic DNA extracted from the patient’s peripheral blood sample was analyzed using a new-generation sequence analysis method (Miseq-Illumina). Genetic screening for sialidosis was performed, and NM_000434,3 p.D135N (c.403G> A) homozygous spot *NEU1* mutation was detected.

To confirm central nervous system and cardiac involvement, the patient underwent brain magnetic resonance imaging (MRI), diffusion MRI, and echocardiography. No pathologies were detected. The patient was prescribed levetiracetam (500 mg 2 × 1) for epilepsy treatment, after which follow-up was conducted in the neurology.

## DISCUSSION

NEU1, a lysosomal glycosidase, catalyzes the breakdown of sialic acids and functions in critical biological pathways. Enzyme activity determined by various *NEU1* mutations and environmental factors affect the symptoms by determining the phenotypic characteristics of the individual^(3)^. In a review conducted in 2019, while myoclonus was reported in all patients with sialidosis type 1, ataxia in 87.8%, and seizures in 73.7%, macular CRS was reported only in 51.2% of the patients^(4)^.

In the data analysis, *NEU1* mutations, which cause more than 40 diseases, are mostly due to missense mutations that do not affect mRNA synthesis^(3)^. The homozygous point mutation causing an amino acid change in NM_000434,3 p.D135N (c.403G> A) in our case is a new pathogenic variant that has not been identified previously. According to the detected mutation in silico (Mutation Taster, PolyPhen-2, Provean, and SIFT) data, it was considered pathogenic with a high probability.

On SD-OCT, the characteristic finding of CRS is increased reflectivity of the inner retinal layers and photoreceptor layer in the fovea^(5)^. Varela et al. measured reflectivity in OCTs of seven patients with sialidosis and galactosialidosis using grayscale analysis (Fiji/ImageJ2) and compared with healthy volunteers. They detected a significant increase in reflectivity in the inner retinal layers compared with the control group^(6)^. Similarly, our case demonstrated hyperreflective nerve fiber layer and ganglion cell layer on which the boundaries could not be clearly determined (Figure 2). In sialidosis, oligosaccharides containing sialic acid accumulate in the ganglion cells^(7)^. As the foveal region is relatively free of ganglion cells, it maintains its redness and causes a CRS appearance. Some studies claim that CRS appearance is caused by increased retinal nerve fiber thickening^(8)^. Similar findings can be found in other metabolic storage diseases. In patients with Niemann-Pick type B, SD-OCT showed hyperreflective areas on the retinal surface, except for the foveal depression area^(9)^.

The hyperreflective appearance on the photoreceptor layer in the foveolar region is attributed to the relatively hyporeflective appearance of the outer retinal layer caused by the increased reflectivity of the inner retinal layers in the vicinity^(10)^.

FFA showed hypofluorescence in the macular region as a result of blockage due to substance accumulation in the ganglion cell layer. This area corresponded to the hyperfluorescent area in SD-OCT. Retinal artery occlusion is among the diseases that should be considered in the differential diagnosis of patients who present with macular CRS. The FFA result in our case was not compatible with retinal artery occlusion.

In this report, we summarized the findings of a patient with type 1 sialidosis and reported a novel mutation that leads to type 1 sialidosis. Genetic mutations identified in type 1 sialidosis cases vary; therefore, the evaluation of both clinical findings and *NEU1* mutations will translate to a better diagnosis. Given that metabolic diseases with macular CRS exhibit a similar phenomenon, SD-OCT findings remain insufficient in the differential diagnosis; thus, genetic mutation screening is vital in this regard.
